# Turkish Adaptation and Psychometric Evaluation of the Tilburg Pregnancy Distress Scale—Revised in Three Independent Gestational Groups

**DOI:** 10.3390/healthcare14152354

**Published:** 2026-08-02

**Authors:** Meryem Kececi Oguzhanoglu, Pelin Mutlu, Kursat Oguzhanoglu, Ebru Ayguler, Busra Seker Atas, Senem Karacabey Cakmak, Ali Cetin

**Affiliations:** Department of Obstetrics and Gynecology, Haseki Training and Research Hospital, University of Health Sciences, 34265 Istanbul, Türkiye; hangunpelin@gmail.com (P.M.); kursatoguzhanoglu@gmail.com (K.O.); ebruayguler@gmail.com (E.A.); bussraseker@hotmail.com (B.S.A.); senemkaracabey@gmail.com (S.K.C.); dralicetin@yahoo.com (A.C.)

**Keywords:** pregnancy distress, Tilburg Pregnancy Distress Scale, psychometrics, confirmatory factor analysis, measurement invariance

## Abstract

**Background/Objectives**: Pregnancy-specific distress is distinct from generic anxiety and depression and predicts adverse perinatal outcomes. This study adapted the 14-item Tilburg Pregnancy Distress Scale—Revised (TPDS-R), which assesses childbirth-related worries, pregnancy-related worries, and partner involvement, into Turkish and evaluated its measurement properties at three defined gestational stages. **Methods**: In this cross-sectional study, 450 pregnant women (150 each at 12, 22, and 32 gestational weeks) completed the Turkish TPDS-R and the Edinburgh Postnatal Depression Scale (EPDS). Adaptation followed an eight-stage procedure including expert panel review and pilot testing. Structural validity, reliability, measurement invariance, and convergent and known-groups validity were examined, with factor analyses estimated by robust diagonally weighted least squares in split-half samples. **Results**: Parallel analysis supported three factors. The three-factor model fitted adequately in the independent confirmatory half (CFI = 0.947) and the total sample (CFI = 0.962), although the TLI fell below the prespecified 0.90 criterion in all three gestational subgroups (0.877–0.899). Internal consistency was acceptable for the total scale (α = 0.70) but weak for pregnancy-related worries (α = 0.60); test–retest reliability was excellent (ICC = 0.94). Only configural invariance was supported. The total score correlated with the EPDS (r = 0.42), and primiparous women reported higher childbirth-related distress (d = 0.47). Pregnancy-related distress was lower in the later gestational groups. A 12-item version omitting items 3 and 10 fitted better but showed weaker external validity. **Conclusions**: The Turkish TPDS-R reproduces the original three-factor structure with excellent temporal stability, supporting dimensional assessment in research and clinical evaluation. Because measurement invariance was not established, between-group differences are descriptive. Criterion-validity studies and cut-off scores are needed before screening use.

## 1. Introduction

Psychological distress is well established to affect both mental and physical health, contributing to the onset of depressive and anxiety disorders and to dysregulation of the neuroendocrine and immune systems [1]. Pregnancy represents a period of heightened vulnerability, during which concerns about fetal health, the partner relationship, and potential complications may precipitate distress. Antenatal anxiety and depression are common: a meta-analysis estimated the prevalence of any antenatal anxiety disorder at approximately 15%, with higher rates in low- and middle-income settings [2], and pregnancy-specific anxiety affects a substantial proportion of women across diverse populations. Fear of childbirth is likewise prevalent, affecting an estimated 14% of pregnant women worldwide [3] and, in a large Turkish sample, reported by as many as 42% [4]. Accumulating evidence links antenatal psychological distress to adverse obstetric outcomes, including preterm birth and reduced likelihood of physiological birth, as well as to cognitive, behavioral, and emotional problems in offspring [5,6].

Importantly, pregnancy-specific distress is increasingly recognized as a construct distinct from generic distress [7,8]. Although pregnancy-specific worries correlate with measures of anxiety and depression, generic instruments do not fully capture them, and pregnancy-specific distress has been associated with adverse maternal and pregnancy outcomes, including pregnancy-induced hypertension, independently of general anxiety [9]. In Turkish populations specifically, fear of childbirth and pregnancy-related worry have been linked to low education, unemployment, unplanned pregnancy, insufficient social support, and limited partner support [10,11], underscoring the cultural relevance of assessing both distress and the partner relationship. These observations support the use of dedicated, pregnancy-specific instruments rather than reliance on generic screening tools alone.

Among such instruments, the Tilburg Pregnancy Distress Scale (TPDS) was developed by Pop et al. through a rigorous protocol based on in-depth interviews with pregnant women, new mothers, midwives, and obstetric nurses [12]. The original 16-item TPDS comprises a partner involvement (PI) dimension and a negative affect (NA) dimension. Cross-cultural examination revealed that, whereas items concerning childbirth and pregnancy worries were psychometrically robust, postpartum-related items showed more variable factor loadings and did not generalize as pregnancy-specific distress symptoms. To address this, Gigase et al. developed the revised scale (TPDS-R), a 14-item instrument comprising a 10-item NA dimension, which includes five-item pregnancy and five-item childbirth subcomponents, together with a four-item PI dimension [13].

In the original longitudinal cohort (*N* = 1081), the TPDS-R demonstrated good test–retest reliability, adequate concurrent validity with the EPDS, good internal consistency (α = 0.81–0.86), and adequate structural validity [13]. Cross-cultural adaptations of pregnancy-specific distress and anxiety instruments have followed broadly comparable psychometric strategies, combining forward and back translation with expert review, factor-analytic evaluation of the dimensional structure, and convergent validation against generic perinatal mood measures. The Pregnancy-Related Anxiety Questionnaire—Revised 2, for example, was developed with formal tests of measurement invariance across parity and across repeated assessments in pregnancy [14]. It has since been adapted and psychometrically evaluated in Turkish [15], German [16], and Chinese [17] samples, with three-factor solutions and acceptable internal consistency reported across settings. A systematic review of pregnancy-related anxiety measurement nevertheless concluded that validation studies frequently lack evidence on measurement invariance and on criterion-referenced cut-off scores [18]. A Greek validation of the original 16-item TPDS (*N* = 196) reported a Cronbach’s alpha of 0.85 and supported the two-factor structure of that instrument [19]. A Persian adaptation of the same instrument yielded a 16-item scale with four factors [20]. Neither examined the revised instrument, which to our knowledge has not been evaluated outside the cohort in which it was developed. Although an earlier Turkish adaptation of the original 16-item TPDS exists [21], the revised 14-item TPDS-R has not previously been validated in a Turkish-speaking obstetric population, nor has its measurement performance been examined separately at distinct gestational stages. This is an important gap, given evidence that the structure and intensity of pregnancy-specific worries shift across gestation.

### Aim and Research Questions

The primary aim of this study was to adapt the TPDS-R into Turkish and to evaluate its measurement properties in a Turkish-speaking obstetric population. The study addressed four research questions:

Does the Turkish TPDS-R reproduce the three-factor structure reported for the original instrument?
Are its internal consistency and three-week temporal stability adequate?
Does it show the expected convergent associations with depressive and anxiety symptoms, and the expected known-groups differences by parity?
Does the instrument function equivalently at 12, 22, and 32 gestational weeks, and are observed differences between these stages interpretable as differences in the underlying construct?

We hypothesized that the Turkish TPDS-R would replicate the original factor structure, demonstrate acceptable internal consistency and temporal stability, show moderate positive correlations with depression and anxiety measures, and discriminate between primiparous and multiparous women.

## 2. Materials and Methods

### 2.1. Study Design and Setting

This cross-sectional methodological study was conducted in the outpatient obstetrics clinic of a tertiary training and research hospital in Istanbul, Türkiye. It was approved by the Clinical Research Ethics Committee of Haseki Training and Research Hospital, University of Health Sciences (approval number 43-2026, dated 4 March 2026). Three independent samples of pregnant women were recruited at three gestational stages (12 ± 1, 22 ± 1, and 32 ± 1 weeks), rather than following a single longitudinal cohort. This design avoids the recall bias inherent in administering identical items to the same women over intervals of several months. Because the three samples comprise different women, all comparisons between gestational stages are between-group comparisons and do not describe within-person change over pregnancy. Translation and cultural adaptation were carried out between 5 and 11 March 2026, with the expert panel convening online on 11 March. The pre-final version was pilot tested between 12 and 15 March. Recruitment for the main study then ran from 16 March to 15 June 2026. Each gestational group was closed once 150 participants had been enrolled. Test–retest administration took place three weeks after enrollment in women recruited between 16 March and 13 April. The study was designed and reported in accordance with the COSMIN recommendations for studies on measurement properties [22] and adhered to the Declaration of Helsinki.

### 2.2. Participants

Eligible participants were pregnant women aged 18 years or older with a singleton pregnancy at the relevant gestational week, literate in Turkish, who provided written informed consent. Exclusion criteria were multiple pregnancy, known fetal anomaly, pre-existing severe psychiatric disorder (schizophrenia, bipolar disorder, or borderline personality disorder), known endocrine disease other than thyroid dysfunction, type 1 diabetes mellitus, substance dependence, and difficulty communicating in Turkish. For confirmatory factor analysis, a minimum of 150 participants per gestational group was targeted. This corresponds to approximately 11 participants per item and therefore satisfies the recommended 10 to 20 participants per item for factor-analytic work [23]. The total sample of 450 women exceeded the minimum of 200 commonly recommended for structural equation modeling. A further 30 pregnant women took part in pilot testing of the pre-final Turkish version and were not included in the main study sample, so that 480 women participated in the study overall.

### 2.3. Measures

Sociodemographic and obstetric form. A researcher-designed form recorded age, education, employment, household income, family type, gestational week, gravidity, parity, history of miscarriage, planned-pregnancy status, psychiatric history, smoking, and body mass index (BMI).

Tilburg Pregnancy Distress Scale—Revised (TPDS-R). The TPDS-R is a 14-item self-report instrument assessing pregnancy-specific distress [13]. Items are rated on a 4-point Likert scale (0 = rarely or never; 1 = sometimes; 2 = fairly often; 3 = very often). The NA dimension (items 1–10) yields scores from 0 to 30, with higher scores indicating greater distress; the PI dimension (items 11–14) is reverse-scored (0–12), with higher scores indicating lower partner involvement. The total score ranges from 0 to 42. The TPDS-R was published under a Creative Commons Attribution license, which permits reuse with attribution; the original publication is cited accordingly [13].

Edinburgh Postnatal Depression Scale (EPDS). The EPDS is a 10-item self-report measure of depressive symptoms over the preceding seven days [24]. Each item is scored 0–3 (total 0–30), with higher scores indicating greater depressive symptomatology. The Turkish validity and reliability study reported a Cronbach’s alpha of 0.79 [25]. The three-item anxiety subscale (EPDS-3A; items 3, 4, and 5) was used as an additional anxiety screen, as supported by prior work demonstrating its sensitivity and specificity for anxiety symptoms in the perinatal period [26,27].

### 2.4. Translation and Cultural Adaptation

The TPDS-R was translated and culturally adapted into Turkish in eight stages, following internationally recommended procedures for the cross-cultural adaptation of patient-reported outcome measures [28,29].

Step 1: Forward translation. Two translators independently rendered the original English instrument into Turkish: an independent professional linguist who was a native Turkish speaker with full English proficiency and no background in health care, and a clinician-researcher (AC), a perinatologist with extensive familiarity with obstetric and psychological research terminology. Both were instructed to produce a natural and readily comprehensible Turkish version appropriate for the general Turkish-speaking population, prioritizing conceptual rather than literal equivalence.

Step 2: Synthesis of forward translations. The two forward translations were reconciled at a meeting attended by the professional translator and three researchers (AC, MKO, SKC), at which the most appropriate rendering of each item was selected, yielding the preliminary Turkish version.

Step 3: Back-translation. The preliminary Turkish version was independently back-translated into English by two authors (KO, EA) with advanced English proficiency who had taken no part in the forward translation stage. Both were unfamiliar with the concepts underlying the questionnaire and were instructed to avoid technical terminology and jargon.

Step 4: Synthesis of back-translations. The two back-translations were reconciled at a further meeting of the professional translator and members of the research team, at which the most appropriate rendering of each item was selected to form the consolidated English back-translation.

Step 5: Review and correction. Where a discrepancy was identified between the back-translation and the original instrument, an iterative process was initiated in which the corresponding forward translation was re-examined and amended. Where identified differences did not warrant an additional translation and review cycle, the process advanced to the next stage.

Step 6: Expert panel review. A multidisciplinary expert panel comprising obstetrician-gynecologists, a perinatologist, a perinatal care nurse, and the professional translator compared the original and back-translated English versions in a collaborative online session. The cultural appropriateness of each translated item was discussed item by item with respect to semantic, idiomatic, experiential, and conceptual equivalence with the original version. Panel members rated the cultural appropriateness of each item on a four-point scale (1 = not relevant at all, 2 = moderately irrelevant, 3 = moderately relevant, 4 = clearly relevant). The predefined criterion for success was the classification of every item as clearly relevant, and the review proceeded iteratively until this criterion was met, at which point the cultural appropriateness of the adapted items was considered established. Because panel ratings were reached collaboratively against this predefined criterion rather than through independent, blinded assessment, formal content validity indices were not computed. Such indices originate in instrument development and presuppose independent expert judgments of newly generated items. They would therefore be uninformative under an iterative consensus procedure in which no new items were generated and the content validity of the source instrument had already been established by its developers.

Step 7: Pilot testing. The pre-final Turkish version was administered face-to-face to a sample of 30 exclusively Turkish-speaking pregnant women attending the antenatal outpatient clinic of the study center. These women were recruited separately and were not included in the main study sample. Eligible women were asked whether they were willing to take part in a study concerning perinatal mental health; those who agreed were informed about the scope of the research and provided written informed consent. Two members of the research team (MKO, PM) subsequently interviewed each participant to identify items that were difficult to understand or to answer and to examine how the items were understood and interpreted. No item was reported as difficult to understand or to answer, and no wording was modified.

Step 8: Adoption of the final version. Summaries of the pilot interviews were reviewed by the researchers at a consultation meeting. The items being considered sufficiently applicable, the final Turkish version of the TPDS-R was adopted for administration.

### 2.5. Procedure

Eligible women who consented completed the sociodemographic form, the Turkish TPDS-R, and the EPDS during routine antenatal care, requiring approximately 15–20 min. For test–retest reliability, a randomly selected subsample of 30 participants per gestational group (total *n* = 90) completed the Turkish TPDS-R again three weeks later. The design consideration that motivated the use of independent gestational samples was the repetition of identical items over intervals of ten and twenty weeks, across which both the meaning of the items and the respondent’s frame of reference change. A fixed three-week interval is, by contrast, a standard psychometric procedure for estimating measurement error. It was chosen to be long enough to limit recall of individual responses yet short enough to preclude meaningful change in gestational context. At the second administration, participants were asked whether any obstetric or medical event had occurred since the first assessment, and their antenatal records were reviewed accordingly. No new diagnoses, hospital admissions, or pregnancy complications were identified in any participant between the two administrations, supporting the assumption of construct stability over this interval.

### 2.6. Statistical Analysis

Descriptive statistics, reliability, and group comparisons were performed in IBM SPSS Statistics version 26.0 (IBM Corp., Armonk, NY, USA) and in Python version 3.11 (Python Software Foundation, Wilmington, DE, USA) using the pandas, numpy, and statsmodels packages. Factor-analytic and measurement invariance models were estimated in R version 4.3.3 (R Foundation for Statistical Computing, Vienna, Austria) using the lavaan package version 0.6-17 and semTools version 0.5-6, with significance set at *p* < 0.05. Continuous variables are presented as mean ± standard deviation (SD) and categorical variables as frequencies and percentages. Questionnaire completeness was verified at the time of administration, and analyses were conducted on complete cases. All questionnaire responses were verified against the original paper questionnaires before scoring. Differences between the three gestational groups in baseline characteristics were examined using one-way ANOVA for continuous variables and the chi-square test for categorical variables. Sampling adequacy was assessed using the Kaiser–Meyer–Olkin (KMO) measure and Bartlett’s test of sphericity.

Because the four-category TPDS-R items are ordinal and show pronounced floor effects, all factor-analytic models were estimated by robust diagonally weighted least squares (WLSMV) applied to the polychoric correlation matrix, and scaled fit indices are reported throughout. Normal-theory maximum-likelihood estimates were computed as a complementary sensitivity analysis for comparability with previous TPDS-R validations and are reported alongside the primary estimates.

The sample was randomly divided into two independent halves using a fixed random seed to ensure reproducibility. Exploratory factor analysis (principal axis factoring with oblimin rotation) was performed on the first half (*n* = 225), with a loading of 0.40 used as a reference threshold (items below it were flagged but retained when theoretically central, to preserve comparability with the source instrument). The number of factors to retain was determined by Horn’s parallel analysis (1000 simulated datasets, 95th percentile criterion) in addition to the Kaiser criterion. Confirmatory factor analysis (CFA) was conducted on the second, independent half (*n* = 225) as the primary test of structural validity, and additionally on the full sample (*N* = 450), which includes the exploratory half and is therefore reported as a precision estimate rather than independent confirmation.

Two models were specified a priori and compared, consistent with the TPDS-R conceptualization in which the negative affect dimension comprises distinct pregnancy and childbirth subcomponents: a two-factor model (negative affect and partner involvement) as a baseline, and the theory-based three-factor model (childbirth, pregnancy, and partner involvement). Model fit was evaluated using χ^2^/df, CFI, TLI, RMSEA with its 90% confidence interval, and SRMR, with CFI/TLI ≥ 0.90 and RMSEA < 0.08 indicating adequate fit [30]. Internal consistency was assessed using four indices: Cronbach’s alpha with 95% bootstrap confidence intervals (2000 resamples); McDonald’s omega computed from the standardized loadings of the confirmatory model; mean inter-item correlations; and corrected item-total correlations computed within each dimension (≥0.30 acceptable). Test–retest reliability was assessed using the intraclass correlation coefficient (ICC; two-way mixed-effects, absolute agreement), interpreted as poor (<0.50), moderate (0.50–0.75), good (0.75–0.90), or excellent (>0.90) [31]. Convergent validity was evaluated using Pearson correlations between TPDS-R and EPDS/EPDS-3A scores. Because the validity comparisons were predefined and hypothesis-driven, *p*-values are reported without correction for multiple testing, and effect sizes with confidence intervals are provided throughout. Known-groups validity was tested using independent-samples *t*-tests (Welch’s correction) with Cohen’s d, comparing primiparous versus multiparous women and women with versus without a psychiatric history; differences between gestational groups were examined using one-way ANOVA with Tukey HSD post hoc tests and eta-squared (η^2^) effect sizes.

Measurement invariance across the three gestational groups was examined for the three-factor model using multi-group categorical confirmatory factor analysis with WLSMV estimation. The sequence appropriate to ordinal indicators was followed. This comprised a configural model, in which the same factor structure was imposed but all parameters were freely estimated within each group; a model additionally constraining item thresholds to equality across groups; and a model additionally constraining factor loadings to equality. Invariance was evaluated using ΔCFI ≤ 0.010 and ΔRMSEA ≤ 0.015. Where an invariance step was not supported, formal partial invariance models were fitted by releasing the constraints on the specific items identified by score tests as most strongly non-invariant, while retaining at least two invariant indicators per factor.

Because the three gestational groups differed in body mass index, employment status, and planned-pregnancy status, between-group differences in TPDS-R scores were additionally examined by analysis of covariance adjusting for these three variables, with partial eta-squared reported as the effect size.

An exploratory sensitivity analysis, which was not prespecified in the study protocol and was undertaken after inspection of the item statistics, examined a 12-item version of the scale omitting items 3 and 10. The 12-item and 14-item versions were compared with respect to model fit, internal consistency, convergent and known-groups validity, and measurement invariance.

Statistical power was estimated for the design as implemented (*n* = 150 per gestational group; *N* = 450). For the three-group one-way analysis of variance, the design provided 80% power to detect an effect of f = 0.147 (η^2^ = 0.021) at a two-sided alpha of 0.05; for two-group comparisons of the sizes examined here, 80% power corresponded to a Cohen’s d of 0.281.

## 3. Results

### 3.1. Sample Characteristics

A total of 450 pregnant women were enrolled in the main study, 150 in each gestational group; a further 30 women participated in pilot testing only. Sociodemographic and obstetric characteristics by gestational group are presented in Table 1. The three groups were comparable in age, parity, and psychiatric history (all *p* > 0.15) but differed in BMI, employment, and planned-pregnancy status (*p* < 0.05). These differences reflect the composition of independent samples recruited at successive gestational stages, and the three variables were therefore carried forward as covariates in the adjusted analyses reported below.

### 3.2. Item Characteristics and Sampling Adequacy

All 14 items showed adequate response distributions, with all four categories used and skewness within acceptable limits (−1.14 to 1.13). Floor rates, defined as the proportion of participants scoring zero on the item, are given in Table 2. They ranged from 26.4% to 51.3% for the ten negative affect items and from 30.2% to 49.6% for the four reverse-scored partner involvement items. Sampling adequacy was acceptable in both the full sample (KMO = 0.749) and the exploratory half (KMO = 0.721), and Bartlett’s test was significant (full sample χ^2^ = 1008.4, *p* < 0.001), indicating the data were adequate, though not optimal, for factor analysis. Item statistics, corrected item-total correlations, and standardized loadings are presented in Table 2. Items 3 and 10 showed comparatively lower item-total correlations (0.27 and 0.23) and standardized loadings (0.34 and 0.32), both below the reference thresholds specified in the Methods section, consistent with their peripheral content within the distress construct.

### 3.3. Structural Validity

**Figure 1:**
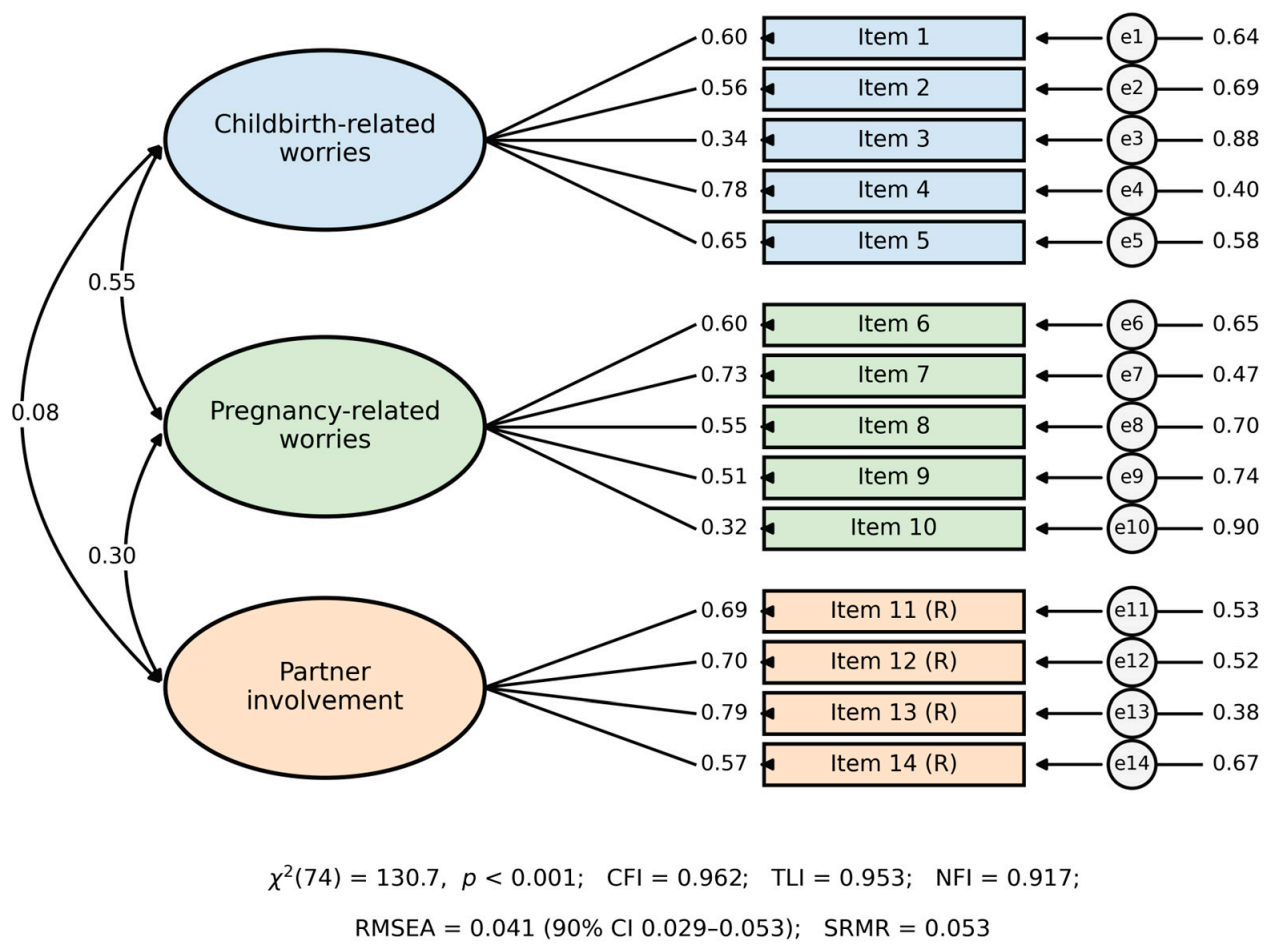
Measurement model of the Turkish TPDS-R. Three-factor measurement model of the Turkish TPDS-R estimated by robust diagonally weighted least squares (WLSMV) in the total sample (*N* = 450). Ellipses denote latent factors and rectangles observed items. Values on single-headed arrows are standardized factor loadings and values on the curved double-headed arrows are factor correlations. Circles labeled e1 to e14 denote item error terms, and the adjacent values are standardized residual variances. Items 11 to 14 are reverse-scored, indicated by (R), so that higher scores on all items denote greater distress or poorer partner involvement.

Exploratory factor analysis (principal axis factoring with oblimin rotation) on the first half (*n* = 225) supported the expected structure. Three eigenvalues exceeded unity (2.99, 2.14, 1.41), accounting for 46.7% of the total variance and 32.1% of common variance after extraction. Childbirth and pregnancy items loaded on their respective negative affect factors, and partner involvement items formed a separable factor without cross-loadings. Primary loadings ranged from 0.18 to 0.71; the lowest again for items 3 and 10. Because the fourth eigenvalue (0.996) fell only marginally below unity, the number of factors was additionally examined by parallel analysis. The first three empirical eigenvalues exceeded the corresponding 95th percentiles of eigenvalues from 1000 random datasets (2.99 versus 1.55; 2.14 versus 1.41; 1.41 versus 1.32), whereas the fourth did not (1.00 versus 1.24), providing independent support for a three-factor solution. Two models were then compared a priori (Table 3). Under WLSMV estimation, the two-factor solution (NA and PI) showed inadequate fit (χ^2^/df = 3.38; CFI = 0.878; TLI = 0.854; RMSEA = 0.073), paralleling the original instrument development [12]. The three-factor model, the a priori specification consistent with the published TPDS-R structure [13], fitted adequately in the independent confirmatory half (*n* = 225), which was the primary test (χ^2^/df = 1.55; CFI = 0.947; TLI = 0.934; RMSEA = 0.050; SRMR = 0.072). It was confirmed with greater precision in the full sample (χ^2^/df = 1.77; CFI = 0.962; TLI = 0.953; RMSEA = 0.041; SRMR = 0.053). Fit was, however, weaker within the individual gestational groups, where the CFI ranged from 0.900 to 0.918 and the TLI remained below 0.90 in all three groups; the 22-week group showed the weakest fit (CFI = 0.900; TLI = 0.877; RMSEA = 0.084). Relative to maximum-likelihood estimation, the categorical estimator improved fit in the full-sample models and in the 22- and 32-week groups but reduced it in the 12-week group (Table 3), so that no gestational group met the TLI criterion under the primary estimator. Model performance was therefore not uniformly adequate across gestational stages. Standardized loadings ranged from 0.32 to 0.79 (Table 2), and the complete measurement model, with standardized loadings, factor correlations, and residual variances, is displayed in Figure 1. The childbirth and pregnancy factors were moderately correlated (r = 0.55), whereas partner involvement correlated negligibly with childbirth-related worries (r = 0.08) and only modestly with pregnancy-related worries (r = 0.30), supporting the relative independence of the partner dimension.

### 3.4. Exploratory Sensitivity Analysis: 12-Item Version

Because items 3 and 10 fell below the prespecified thresholds for both corrected item-total correlation and standardized loading, an exploratory sensitivity analysis examined a 12-item version omitting these two items. The 12-item three-factor model fitted better than the 14-item model at every level of analysis. The values were CFI = 0.983, TLI = 0.978, RMSEA = 0.033 and SRMR = 0.044 in the total sample (χ^2^/df = 1.47), and CFI = 0.971, TLI = 0.962 and RMSEA = 0.043 in the independent confirmatory half. Within the gestational groups the CFI was 0.934, 0.929, and 0.951 at 12, 22, and 32 weeks, respectively. Notably, the Tucker–Lewis index reached the prespecified criterion in all three gestational groups under the 12-item specification (0.915, 0.909, and 0.936), which it had not under the 14-item model. Internal consistency of the 12-item total score was equivalent to that of the 14-item total (α = 0.698, 95% CI 0.645–0.738 versus α = 0.695, 95% CI 0.644–0.738), and the mean inter-item correlation was slightly higher (0.163 versus 0.142). The improvement in internal structure was, however, accompanied by weaker external validity. The correlation of the total score with the EPDS fell from 0.422 to 0.395 and that of the negative affect dimension from 0.322 to 0.283, while the parity difference in childbirth-related worries fell from d = 0.47 to d = 0.41. Measurement invariance was also unaffected: constraining item thresholds produced the same deterioration under the 12-item model as under the 14-item model (ΔCFI = −0.052 in both), and constraining loadings produced a slightly larger one (ΔCFI = −0.022 versus −0.015). The 14-item version is therefore retained as the primary specification, both to preserve comparability with the source instrument and with other adaptations and because the shorter form, although structurally cleaner, does not improve the external validity of the scale or resolve its principal limitation.

### 3.5. Measurement Invariance Across Gestational Groups

Measurement invariance of the three-factor model across the three gestational groups was examined in a stepwise manner appropriate to ordinal indicators (Table 4). The configural model, in which the same factor structure was imposed but all parameters were freely estimated within each group, met the prespecified fit criterion (CFI = 0.909, RMSEA = 0.066), indicating that the three-factor configuration was tenable at each gestational stage. Constraining item thresholds to equality across groups produced a marked deterioration in fit (ΔCFI = −0.052), substantially exceeding the prespecified ≤0.010 criterion, and additionally constraining factor loadings produced a further deterioration (ΔCFI = −0.015). Formal partial invariance models were therefore fitted. Score tests identified items 8, 7, 2, and 1 as the most strongly non-invariant indicators. Releasing the threshold constraints on items 7 and 8 reduced the discrepancy relative to the configural model to ΔCFI = −0.036, and releasing the constraints on all four items reduced it to ΔCFI = −0.021; releasing the corresponding loadings in addition produced no further improvement (ΔCFI = −0.024). Even under these formal partial models, therefore, the discrepancy remained above the prespecified criterion, and partial threshold or loading invariance could not be established. Taken together, these results support a common factor configuration across gestational stages but do not support equivalence of item thresholds or loadings. Differences in observed scores between gestational groups must accordingly be interpreted as descriptive comparisons of observed scores and cannot be interpreted as equivalent differences in the underlying latent construct.

### 3.6. Reliability

Internal consistency, McDonald’s omega, mean inter-item correlations, and their confidence intervals are presented in Table 5. Internal consistency was acceptable for the total scale (α = 0.695, 95% CI 0.644–0.738) and for the negative affect and partner involvement dimensions (α = 0.703 and 0.689, respectively), but was modest for the two negative affect subcomponents, particularly pregnancy-related worries (α = 0.601, 95% CI 0.520–0.666). The lower bound of this interval falls well below conventional standards, and the internal consistency of this subcomponent should therefore be regarded as a limitation of the Turkish adaptation rather than as unequivocally acceptable. McDonald’s omega, which does not assume tau-equivalence, was somewhat higher than alpha for each dimension (0.728, 0.679, and 0.781). Mean inter-item correlations for the three dimensions (0.235 to 0.362) fell within or close to the range generally considered optimal, whereas the value for the 14-item total score (0.142) reflects the multidimensional composition of the total scale. Test–retest reliability over a three-week interval (*n* = 90) was excellent (ICC = 0.94, 95% CI 0.91–0.96, *p* < 0.001), indicating strong temporal stability.

### 3.7. Inter-Item Correlations

**Figure 2:**
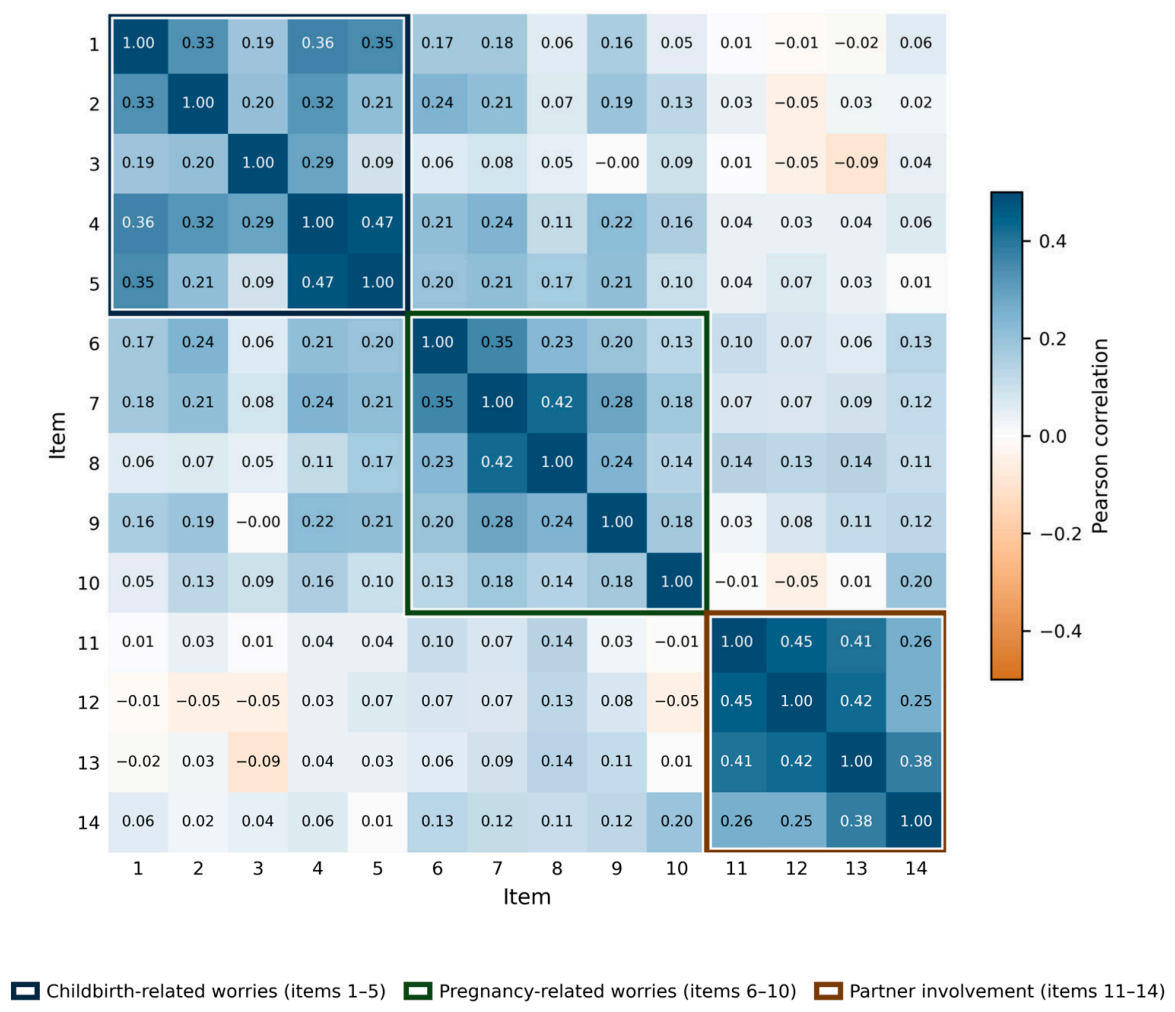
Inter-item correlation matrix of the Turkish TPDS-R. Pearson correlations between the 14 TPDS-R items (*N* = 450). The matrix is symmetric, and the diagonal is fixed at unity. Items 11 to 14 are reverse-scored, so that higher values on all items indicate greater distress or poorer partner involvement. Outlined blocks mark the three dimensions, in the colors used in Figure 1. The diverging color scale is centered at zero and truncated at ±0.50, with positive correlations shown in blue and negative correlations in orange; correlations are printed within each cell.

The full matrix of inter-item correlations is presented in Figure 2. Correlations were consistently higher within than between dimensions. Items 3 and 10 showed the weakest associations with the other items of their respective dimensions (item 3 with items 1, 2, 4, and 5: r = 0.09 to 0.29; item 10 with items 6 to 9: r = 0.13 to 0.18). The partner involvement items, by contrast, were strongly interrelated (r = 0.25 to 0.45) and largely uncorrelated with the negative affect items.

### 3.8. Convergent Validity

The TPDS-R total score correlated moderately and positively with the EPDS total score (r = 0.42, *p* < 0.001) and with the EPDS-3A anxiety subscale (r = 0.35, *p* < 0.001). The negative affect dimension correlated with the EPDS (r = 0.32, *p* < 0.001) and the partner involvement dimension correlated with the EPDS (r = 0.34, *p* < 0.001). These associations provide initial convergent evidence, in the direction and of the magnitude expected if pregnancy-specific distress overlaps with, but remains distinct from, generic depressive and anxiety symptoms. They do not, however, constitute a comprehensive assessment of convergent and discriminant validity: the EPDS-3A is a three-item subscale of the same instrument rather than an independent anxiety measure, and no pregnancy-specific comparator was administered.

### 3.9. Known-Groups Validity and Differences Between Gestational Groups

**Figure 3:**
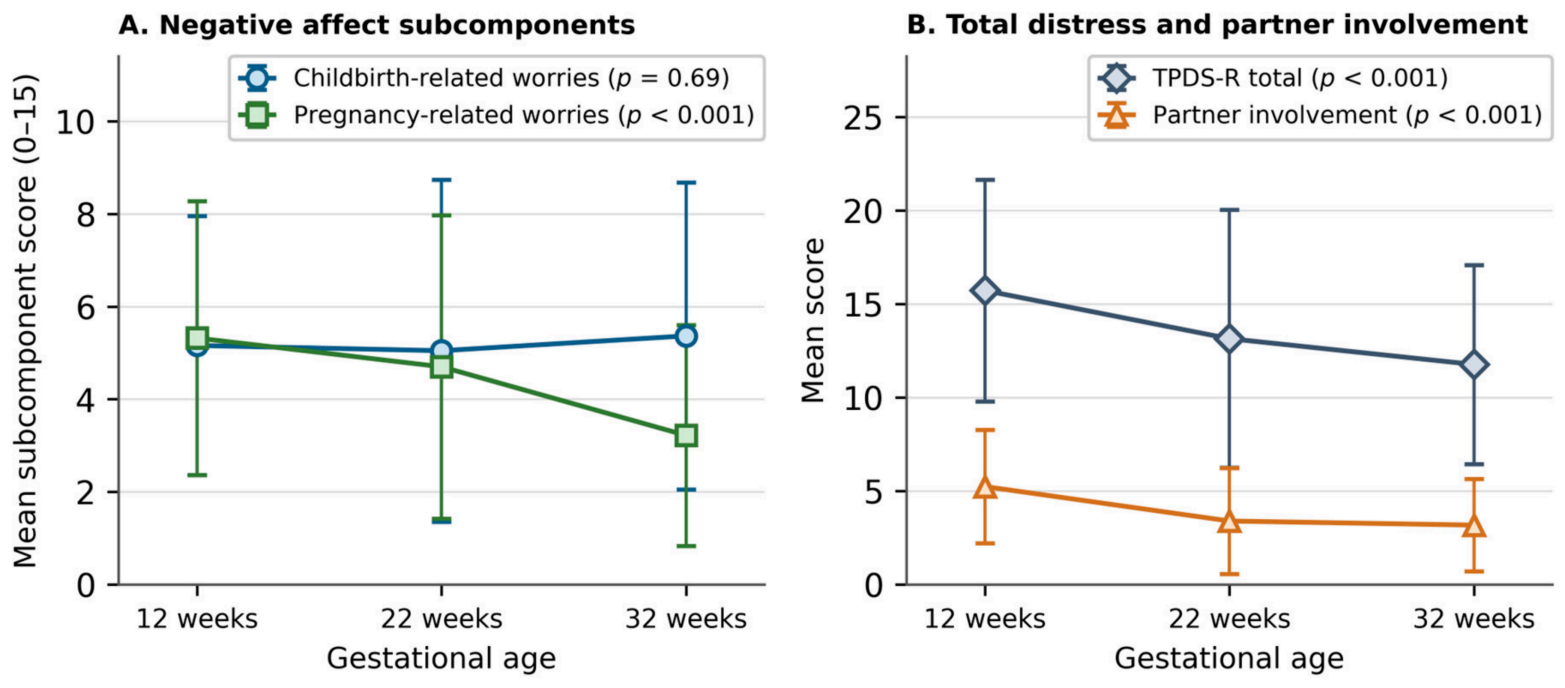
Mean TPDS-R scores by gestational group. (**A**) Negative affect subcomponents: pregnancy-related worry scores are lower in the later gestational groups, whereas childbirth-related worry scores do not differ between groups. (**B**) Total distress and partner involvement, both lower in the later gestational groups; because the partner involvement dimension is reverse-scored, its lower score indicates more favorable perceived partner involvement. *p*-values are from one-way analysis of variance and are shown in the legend of each panel. Error bars represent standard deviations. Because the three gestational samples comprise different women, the figure displays differences between independent groups and not change within individual pregnancies.

Primiparous women reported higher childbirth-related distress than multiparous women (5.9 ± 3.4 vs. 4.4 ± 2.9; t = 4.97, *p* < 0.001, d = 0.47, 95% CI 0.28 to 0.65), whereas pregnancy-related distress did not differ by parity (*p* = 0.85). Women with a self-reported psychiatric history reported higher total distress (18.0 ± 8.0 vs. 13.4 ± 6.2; d = 0.74, 95% CI 0.22 to 1.26); with only 15 such participants, this comparison should be regarded as exploratory and the confidence interval is correspondingly wide. Across gestational groups, total distress scores were lower in the later gestational groups (15.7, 13.1, and 11.8 at 12, 22, and 32 weeks; F = 16.36, *p* < 0.001, η^2^ = 0.068; Tukey HSD: 12 versus 22 and 12 versus 32 weeks, both *p* < 0.001). This pattern was accounted for by the pregnancy subcomponent (5.3, 4.7, and 3.2; F = 20.84, *p* < 0.001, η^2^ = 0.085), whereas the childbirth subcomponent did not differ between groups (5.2, 5.0, and 5.4; F = 0.37, *p* = 0.69). Partner involvement scores were also lower in the two later gestational groups than in the 12-week group (F = 24.70, *p* < 0.001, η^2^ = 0.100). Because the three gestational samples comprise different women, these are differences between independent groups assessed at different gestational weeks and do not describe change within individual pregnancies. Dimension and total scores by group are presented in Table 6 and Figure 3.

After adjustment for body mass index, employment status, and planned-pregnancy status, the differences between gestational groups persisted for pregnancy-related worries, partner involvement, and the total score, with modestly attenuated effect sizes. The difference in the negative affect composite became borderline (*p* = 0.050), and the childbirth subcomponent remained non-significant (Table 6). The compositional differences between the independent gestational samples therefore do not account for the observed pattern of scores.

## 4. Discussion

This study adapted the TPDS-R into Turkish and evaluated its measurement properties in 450 pregnant women recruited at three defined gestational stages. The instrument reproduced the three-factor structure of the original scale, with adequate fit in an independent confirmatory sample and in the total sample, and showed excellent test–retest reliability and coherent convergent and known-groups validity. Internal consistency was acceptable for the total scale but modest for the two negative affect subcomponents, and model fit was not uniformly adequate within the individual gestational groups. To our knowledge, this is the first validation of the revised 14-item TPDS-R in a Turkish-speaking obstetric population and the first to examine its performance separately at three defined gestational stages.

The central structural finding was that a three-factor model, distinguishing childbirth-related worries, pregnancy-related worries, and partner involvement, fitted the data adequately whereas a two-factor model did not. This reproduces the experience of the original developers, who resolved an inadequate two-component solution by modeling subcomponents of the negative affect dimension; our specification was therefore an a priori alignment with the established structure rather than a post hoc modification. The three-factor solution was supported both by parallel analysis in the exploratory half and by adequate fit in the independent confirmatory half. This support should nevertheless be qualified. Within the individual gestational groups the Tucker–Lewis index remained below the prespecified threshold of 0.90 in all three groups, and the 22-week group additionally showed an RMSEA above 0.08. The dimensional architecture of the instrument therefore appears to be reproduced at the level of the full sample, but its performance was not consistently adequate at every gestational stage, and the structure should be regarded as supported rather than firmly established in this population. Replication in an independent linguistic and cultural context nonetheless strengthens confidence in the general dimensional architecture of the instrument. The Greek and Persian adaptations concern the original 16-item TPDS and reported two- [19] and four-factor [20] solutions respectively. They therefore cannot corroborate the three-factor structure of the revised scale.

Equal group sizes of 150 mean that sample size cannot explain why fit was weakest specifically at 22 weeks. All three within-group models nevertheless sit at the low end of what this specification requires: 150 participants corresponds to approximately 11 per item, at the lower bound of the 10 to 20 per item generally recommended for factor-analytic work [23]. Several substantive explanations merit consideration. The second trimester follows the routine anomaly scan, after which the content of pregnancy-related worry may be reorganized around newly salient concerns rather than around the general apprehension captured by the original items. In addition, the boundary between pregnancy-related and childbirth-related worry may be least distinct at mid-gestation, when delivery is neither imminent nor remote, which would reduce the discriminability of the two negative affect subfactors. The two weakest items, items 3 and 10, also behaved less consistently in this group. A further consideration concerns estimation. Under maximum likelihood the 12-week group had shown the best fit of the three, whereas under the categorical estimator its indices fell to the level of the other two groups. Categorical estimation is based on polychoric correlations and estimated thresholds, and where some response categories are sparsely endorsed these quantities are less precisely determined; the apparent advantage of the 12-week group under maximum likelihood therefore appears to have been partly an artifact of treating ordinal, floor-affected items as continuous. These explanations are post hoc and require testing in independent samples, but taken together they indicate that model fit is modest across all three gestational groups rather than selectively poor at 22 weeks.

Convergent validity was supported by a moderate correlation between the TPDS-R total and the EPDS (r = 0.42), consistent with the original cohort (negative affect–EPDS 0.40–0.52) and directionally consistent with the value of 0.27 reported for the Persian adaptation of the original instrument [20]. This reflects the expected partial overlap between pregnancy-specific distress and generic depressive symptoms, while the retention of distinct, pregnancy-specific content supports a dedicated instrument rather than generic measures alone. The correlation with the EPDS-3A anxiety subscale (r = 0.35) is congruent with this interpretation, but its evidential value is limited: the EPDS-3A comprises three items drawn from the same instrument rather than an independent anxiety measure, and no pregnancy-specific measure of anxiety or distress was administered. The present findings therefore constitute initial convergent evidence rather than a comprehensive evaluation of convergent and discriminant validity, and the weak association between partner involvement and the negative affect dimensions provides only indirect evidence of divergence.

Internal consistency warrants careful rather than reassuring interpretation. The alpha coefficients ranged from 0.60 to 0.70. Although alpha is known to depend on the number of items, and short subscales of four or five items systematically yield lower coefficients even when items are coherent [32], this consideration does not by itself render all dimensions unequivocally acceptable. The pregnancy-related worries subcomponent in particular had an alpha of 0.601 with a lower confidence bound of 0.520, which falls below any conventional standard and should be treated as a limitation of the Turkish adaptation. McDonald’s omega, which does not assume tau-equivalence, was somewhat more favorable (0.679 to 0.781), and mean inter-item correlations for the three dimensions fell within or close to the optimal range. The pronounced floor effect on the negative affect items reported above restricted variance and contributes to the modest coefficients, implying that the scale discriminates most reliably at higher distress levels. The sample was drawn from the routine antenatal clinic of a tertiary center and therefore comprises a mixed obstetric population, including both uncomplicated pregnancies followed locally and referred higher-risk pregnancies. The floor rates observed here should not be read as characteristic of either a community or a high-risk sample alone. The excellent test–retest stability (ICC = 0.94) indicates that measurement was stable over the three-week interval, and that the modest alphas reflect item heterogeneity rather than unreliable measurement over time. Given that no obstetric or medical events were recorded between administrations, this is also consistent with stability of the construct itself. Interpretation of a single total score also requires caution, since the partner involvement dimension correlated only weakly with the two negative affect dimensions. The total score should therefore be understood as a summary index of a multidimensional construct rather than as a measure of a single homogeneous trait, and the dimension scores are likely to be more informative in most applications.

An earlier Turkish adaptation of the original 16-item TPDS reported a total alpha of 0.83 in a community sample of 275 women, with 0.72 for its five-item partner involvement subscale [21]. Our estimate for the four partner involvement items retained in the revised instrument (0.69) is closely consistent with that value, whereas internal consistency of the negative affect dimension was lower here (0.70 versus 0.83), which is expected given that the revision removed the postpartum and social items that had broadened the content of the original subscale. The two adaptations are therefore complementary, covering the original and the revised instrument respectively.

Items 3 and 10 fell below the prespecified thresholds for both corrected item-total correlation and standardized loading, and their performance should be acknowledged as a psychometric limitation of the Turkish adaptation. Their weakness appears conceptually intelligible rather than attributable to translation failure. Item 3, which concerns thinking about different modes of delivery, indexes cognitive preoccupation rather than emotional distress. In a setting where mode of delivery is a routine and widely discussed decision, frequent consideration of delivery options may represent ordinary preparatory reasoning rather than worry, which would attenuate its association with the affectively loaded remaining items. Item 10, which concerns whether one is maintaining a sufficiently healthy lifestyle, may in the Turkish context be understood as a normative self-appraisal against expectations of maternal conduct rather than as a source of apprehension. Both interpretations are consistent with the observed pattern of inter-item correlations. Importantly, content validity and structural validity are distinct properties: the expert panel judged both items to be culturally relevant to the construct, and this judgment is not contradicted by the finding that the items discriminate weakly in this sample. The exploratory 12-item version omitting these items fitted markedly better at every level of analysis, and under this specification the Tucker–Lewis index met the prespecified criterion in all three gestational groups, which it had not for the full scale. Three considerations nevertheless weigh against adopting the shorter form here. First, the gain in internal structure was accompanied by a loss of external validity, since correlations with depressive symptoms and the parity difference in childbirth-related worries were both attenuated. This indicates that items 3 and 10, although weakly related to the remaining items, carry variance that is systematically related to constructs outside the scale. Second, the shorter form did not resolve the principal limitation of the instrument in this sample: the deterioration in fit on constraining item thresholds across gestational groups was identical for the two versions, and slightly greater for loadings, so measurement invariance remained unsupported. Third, items 3 and 10 were identified within the present dataset, so part of the apparent improvement in fit reflects capitalization on sample-specific variation and would require replication in an independent sample to be interpreted as a genuine structural advantage. The 14-item version has therefore been retained as the primary specification, and the 12-item structure is reported as an exploratory finding that warrants formal confirmatory evaluation in an independent sample rather than as a recommended alternative.

The known-groups findings provide additional construct-validity evidence congruent with the original cohort and the broader literature. Primiparous women reported higher childbirth-related distress than multiparous women, with no parity difference in pregnancy-related worry, a theoretically meaningful dissociation: first-time mothers, lacking direct experience of labor, face greater uncertainty about delivery, whereas worry about the ongoing pregnancy and the baby’s health is common regardless of parity. This accords with Turkish studies identifying nulliparity, limited birth preparation, and prior negative birth experience as predictors of fear of childbirth, and with international evidence of higher childbirth fear in primiparous women [33].

The comparison of scores across gestational stages requires particular care. Total distress scores were lower in the later gestational groups, and this pattern was accounted for entirely by the pregnancy subcomponent, whereas childbirth-related worry did not differ between groups (Figure 3). Because the three samples comprise different women assessed at a single time point, these are differences between independent groups and not trajectories within individual pregnancies; the design cannot establish whether any individual woman’s distress changes over gestation. The lower pregnancy-related worry scores in the later groups are plausibly related to the reassurance provided by routine antenatal surveillance, particularly confirmation of fetal viability by early ultrasonography [34], whereas anticipatory concern about the approaching delivery is sustained as term nears [35]. Two further constraints bear on the interpretation of these differences. First, measurement invariance across gestational groups was supported only at the configural level; neither threshold nor loading invariance was established, and formal partial invariance models releasing the four most strongly non-invariant items did not bring the discrepancy within the prespecified criterion. Part of the observed between-group difference may therefore reflect stage-specific differences in how particular items are endorsed rather than differences in the underlying construct, and the observed differences cannot be interpreted as equivalent latent-mean differences. Second, the three groups differed in body mass index, employment, and planned-pregnancy status. Rather than assuming that these compositional differences would attenuate the observed pattern, we tested this directly: after adjustment, the differences in pregnancy-related worry, partner involvement, and the total score persisted with modestly reduced effect sizes, whereas the negative affect composite became borderline. Compositional differences therefore do not account for the observed pattern, but the invariance findings remain the principal constraint on its interpretation.

The partner involvement dimension also merits comment. Perceived partner involvement was least favorable in the 12-week group and more favorable in the later gestational groups, and was only weakly correlated with the negative affect factors, confirming its relative independence as a distinct, relationally oriented domain rather than a facet of distress. This is particularly relevant in the Turkish context, where partner and broader social support are repeatedly identified as protective against fear of childbirth and antenatal distress [36,37]. Capturing this dimension alongside negative affect enhances the instrument’s value, enabling identification of women whose distress may be compounded by limited relational support and who might benefit from partner-engaging interventions.

The implications for practice should be stated conservatively. The present findings support the use of the Turkish TPDS-R for dimensional assessment of pregnancy-specific distress in research and in clinical evaluation. Its capacity to distinguish domains of worry may help clinicians tailor supportive responses, for example structured birth-preparation education where childbirth-related worry predominates, and reassurance and complication-focused counseling where pregnancy-related worry predominates. The study does not, however, establish clinical cut-off scores, sensitivity, specificity, predictive values, receiver operating characteristic performance, or criterion validity against a structured diagnostic assessment. The instrument therefore cannot at present be recommended for use as a screening tool or for the identification of individual women with clinically elevated distress, and the distinction between dimensional assessment, research use, and clinical screening should be maintained. Recommendations regarding screening applications must await criterion-validity studies and the establishment of clinically meaningful thresholds [38].

The principal strengths include a substantial sample (*N* = 450) that exceeds most prior TPDS adaptations and satisfies the requirements for confirmatory factor analysis, together with stratified recruitment across three defined gestational windows, which permitted stage-specific evaluation. The adaptation itself was thorough, comprising eight stages and incorporating an independent professional translator, blinded back-translation, multidisciplinary expert panel review, and pilot testing in a separate sample. Further strengths are the use of an estimator appropriate to the ordinal, floor-affected response format, the formal testing of measurement invariance including partial invariance models, and excellent test–retest reliability.

Several limitations should be acknowledged. First, internal consistency, while acceptable for the total scale, was modestly lower than in the original Dutch cohort (0.81–0.86) and was weak for the pregnancy-related worries subcomponent. Second, the cross-sectional design, chosen to avoid recall bias over long intervals, precludes evaluation of longitudinal measurement invariance within individuals and of any within-person change across gestation. Third, recruitment from a single tertiary referral center in one metropolitan area may limit generalizability. Because tertiary centers receive referrals of higher-risk pregnancies and serve a socioeconomically distinctive urban population, the sample may over-represent women with complex obstetric histories and under-represent rural and lower-resource settings. The floor effects observed here may therefore differ in community samples. Fourth, model fit was not uniformly adequate across gestational groups, and measurement invariance was established only at the configural level, so between-group score comparisons remain descriptive. Fifth, convergent validity was examined only against the EPDS and its brief anxiety subscale, and a formal test of discriminant validity was not included. Finally, criterion validity against a structured diagnostic interview and the establishment of clinical cut-off scores were beyond this study’s scope.

Future research should establish clinically meaningful cut-off scores against structured diagnostic interviews, evaluate criterion and predictive validity for adverse maternal and neonatal outcomes, and examine longitudinal measurement invariance and responsiveness to change, since the capacity of the instrument to detect clinically meaningful change over time has not yet been demonstrated. Formal confirmatory evaluation of the 12-item structure, and studies in community and rural samples and in samples incorporating partner-reported data, would further clarify the generalizability and clinical applications of the present findings.

## 5. Conclusions

The Turkish version of the TPDS-R reproduces the three-factor structure of the original scale, demonstrates excellent temporal stability, and shows theoretically coherent associations with depression and anxiety measures as well as the expected difference by parity. Internal consistency was acceptable for the total scale but modest for the negative affect subcomponents, and model fit was not uniformly adequate across gestational stages. Measurement invariance across gestational groups was supported only at the configural level; neither threshold nor loading invariance was established, including under formal partial invariance models. Differences between gestational groups must therefore be interpreted as descriptive comparisons of observed scores rather than as equivalent latent-mean differences. The instrument can be recommended for dimensional assessment of pregnancy-specific distress in research and clinical evaluation in Turkish-speaking antenatal settings, whereas its use as a screening instrument will require criterion-validity studies and validated, population-specific cut-off scores. An exploratory 12-item version omitting two weakly performing items fitted better at every level of analysis and merits formal confirmatory evaluation.

## Figures and Tables

**Table 1:** Sociodemographic and obstetric characteristics by gestational group.

Characteristic	12 Weeks (*n* = 150)	22 Weeks (*n* = 150)	32 Weeks (*n* = 150)	*p*
Age, years, mean ± SD	29.5 ± 4.9	29.7 ± 5.4	29.0 ± 5.0	0.474
BMI, kg/m^2^, mean ± SD	26.7 ± 4.9	26.9 ± 3.5	30.3 ± 4.2	<0.001
Employed, *n* (%)	60 (40.0)	37 (24.7)	21 (14.0)	<0.001
Planned pregnancy, *n* (%)	124 (82.7)	118 (78.7)	103 (68.7)	0.013
Psychiatric history, *n* (%)	3 (2.0)	6 (4.0)	6 (4.0)	0.538

BMI, body mass index; SD, standard deviation. *p*-values are from one-way ANOVA (continuous variables) and the chi-square test (categorical variables).

**Table 2:** Item descriptive statistics, corrected item-total correlations, and standardized factor loadings (*N* = 450).

Item	Mean (SD)	Skewness	Item-Total r	Loading (WLSMV)	Floor, %
1. I worry about childbirth	1.20 (0.99)	0.51	0.46	0.60	26.4
2. I get very tense from what I hear about childbirth	1.04 (1.01)	0.60	0.39	0.56	38.0
3. I often think about different ways of childbirth	0.84 (1.05)	0.96	0.27	0.34	51.3
4. I am afraid I will lose self-control during childbirth	1.01 (0.96)	0.61	0.56	0.78	36.4
5. Childbirth is troubling me	1.10 (1.04)	0.59	0.41	0.65	34.2
6. I get very tense about pregnancy complications	0.86 (0.97)	0.84	0.35	0.60	46.4
7. I worry about the pregnancy	0.86 (0.97)	0.85	0.49	0.73	46.9
8. I worry about the health of my baby	0.91 (0.98)	0.81	0.40	0.55	43.1
9. I get very tense during a pregnancy check-up	0.72 (0.87)	1.13	0.34	0.51	50.0
10. I worry about a healthy enough lifestyle	1.07 (1.07)	0.67	0.23	0.32	37.1
11. My partner and I enjoy the pregnancy together	1.83 (0.98)	−0.34	0.49	0.69	30.2
12. The pregnancy brought my partner and I closer	2.02 (1.02)	−0.69	0.49	0.70	41.8
13. I feel supported by my partner	2.22 (0.96)	−1.14	0.55	0.79	49.6
14. I can really share my feelings with my partner	1.98 (1.12)	−0.62	0.38	0.57	45.8

SD, standard deviation. Item-total r, corrected item-total correlation computed within the dimension to which the item belongs. Loading, standardized factor loading from the three-factor confirmatory model estimated by robust diagonally weighted least squares (WLSMV). Items 11–14 (partner involvement) are positively worded and reverse-scored before summation, such that higher dimension scores indicate poorer partner involvement; the item means shown here reflect raw (unreversed) responses. Floor, percentage of participants scoring zero on the item after reverse-scoring where applicable.

**Table 3:** Confirmatory factor analysis fit indices.

Model/Sample	χ^2^/df	CFI	TLI	RMSEA	SRMR	CFI (ML)	TLI (ML)	RMSEA (ML)
Two-factor (NA, PI), total	3.38	0.878	0.854	0.073	0.076	0.820	0.784	0.070
Three-factor, independent half (*n* = 225)	1.55	0.947	0.934	0.050	0.072	0.909	0.888	0.050
Three-factor, total (*N* = 450)	1.77	0.962	0.953	0.041	0.053	0.935	0.920	0.043
Three-factor, 12 weeks (*n* = 150)	1.39	0.903	0.880	0.051	0.081	0.956	0.943	0.030
Three-factor, 22 weeks (*n* = 150)	2.06	0.900	0.877	0.084	0.098	0.865	0.825	0.092
Three-factor, 32 weeks (*n* = 150)	1.53	0.918	0.899	0.060	0.090	0.886	0.853	0.068
Three-factor, 12-item version, total	1.47	0.983	0.978	0.033	0.044	0.969	0.959	0.034
Three-factor, 12-item version, independent half	1.42	0.971	0.962	0.043	0.064	–	–	–

NA, negative affect; PI, partner involvement; CFI, comparative fit index; TLI, Tucker–Lewis index; RMSEA, root mean square error of approximation; SRMR, standardized root mean square residual. Primary estimates (columns two to seven) are from robust diagonally weighted least squares (WLSMV) applied to the polychoric correlation matrix, with scaled indices reported. The final three columns give the corresponding indices obtained under normal-theory maximum likelihood (ML), presented as a complementary sensitivity analysis. Thresholds for adequate fit: CFI/TLI ≥ 0.90; RMSEA < 0.08. The 12-item version omits items 3 and 10 and was examined as an exploratory, non-prespecified sensitivity analysis.

**Table 4:** Measurement invariance of the three-factor model across the three gestational groups.

Model	χ^2^	df	CFI	RMSEA	ΔCFI	ΔRMSEA
Configural	368.2	222	0.909	0.066	–	–
Threshold invariance (all items)	503.3	272	0.857	0.076	−0.052	+0.010
Loading invariance (all items)	549.0	294	0.842	0.076	−0.015	0.000
Partial threshold invariance (items 7, 8 free)	464.8	260	0.873	0.073	−0.036	+0.007
Partial threshold invariance (items 1, 2, 7, 8 free)	427.4	248	0.889	0.070	−0.021	+0.004
Partial loading invariance (items 1, 2, 7, 8 free)	455.6	270	0.885	0.068	−0.024	+0.002

CFI, comparative fit index; RMSEA, root mean square error of approximation. All models were estimated by multi-group categorical confirmatory factor analysis with robust diagonally weighted least squares (WLSMV) across the three independent gestational groups (*n* = 150 each). For the two sequential models, Δ values are relative to the preceding, less constrained model; for the partial invariance models, Δ values are relative to the configural model. Invariance was judged by ΔCFI ≤ 0.010 and ΔRMSEA ≤ 0.015. Neither full nor partial threshold or loading invariance was supported.

**Table 5:** Internal consistency and homogeneity of the Turkish TPDS-R (*N* = 450).

Scale	No. of Items	Cronbach’s α	95% CI	McDonald’s ω	Mean Inter-Item r
Childbirth-related worries	5	0.657	0.592–0.711	0.728	0.280
Pregnancy-related worries	5	0.601	0.520–0.666	0.679	0.235
Negative affect	10	0.703	0.648–0.746	–	0.194
Partner involvement	4	0.689	0.630–0.737	0.781	0.362
Total scale (14 items)	14	0.695	0.644–0.738	–	0.142
Total scale (12-item version)	12	0.698	0.645–0.738	–	0.163

CI, confidence interval. Confidence intervals for Cronbach’s alpha are 95% bootstrap intervals based on 2000 resamples. McDonald’s omega was computed from the standardized loadings of the three-factor confirmatory model and is reported for the three latent dimensions only, since the negative affect composite and the total score span more than one factor. Test–retest reliability over three weeks (*n* = 90) was ICC = 0.94 (95% CI 0.91–0.96).

**Table 6:** TPDS-R dimension and total scores by gestational group, unadjusted and adjusted.

Dimension (Range)	12 Weeks	22 Weeks	32 Weeks	Unadjusted *p* (η^2^)	Adjusted *p* (η^2^p)
Childbirth (0–15)	5.2 ± 2.8	5.0 ± 3.7	5.4 ± 3.3	0.694 (0.002)	0.337 (0.005)
Pregnancy (0–15)	5.3 ± 3.0	4.7 ± 3.3	3.2 ± 2.4	<0.001 (0.085)	<0.001 (0.069)
Negative affect (0–30)	10.5 ± 4.7	9.7 ± 5.9	8.6 ± 4.7	0.006 (0.023)	0.050 (0.013)
Partner involvement (0–12)	5.2 ± 3.0	3.4 ± 2.8	3.2 ± 2.5	<0.001 (0.100)	<0.001 (0.074)
Total (0–42)	15.7 ± 5.9	13.1 ± 6.9	11.8 ± 5.3	<0.001 (0.068)	<0.001 (0.045)

Values are mean ± standard deviation. Unadjusted *p*-values and eta-squared (η^2^) effect sizes are from one-way ANOVA. Adjusted *p*-values and partial eta-squared (η^2^p) are from analysis of covariance additionally including body mass index, employment status, and planned-pregnancy status as covariates. Because measurement invariance across gestational groups was not established, all between-group comparisons are descriptive comparisons of observed scores.

## Data Availability

The data presented in this study are available on request from the corresponding author. The data are not publicly available because they comprise individual-level psychological and obstetric data collected at a single center, including responses to an item concerning self-harm ideation. Participants consented to participation in this study and not to public deposition of their data, the institutional ethics approval does not extend to open data sharing, and the combination of sociodemographic, obstetric and clinical variables could permit re-identification within this sample.
